# Insulin-like growth factor 1 (IGF-1)-induced changes in cardiac inducible nitric oxide synthase (iNOS) in obese rats

**DOI:** 10.3389/fendo.2025.1716392

**Published:** 2026-01-16

**Authors:** Sonja Zafirovic, Milan Obradovic, Katarina Banjac, Emina Sudar-Milovanovic, Anastasija Pajcin, Jelena Radovanovic, Esma R Isenovic

**Affiliations:** Department of Radiobiology and Molecular Genetics, VINČA Institute of Nuclear Sciences - National Institute of the Republic of Serbia, University of Belgrade, Belgrade, Serbia

**Keywords:** heart, IGF-1, iNOS, NO, obesity

## Abstract

**Introduction:**

The role of insulin-like growth factor 1 (IGF-1) in preserving cardiovascular (CV) health, a well-established fact, cannot be overstated. IGF-1 affects inducible nitric oxide synthase (iNOS) activity, contributing to metabolic homeostasis by promoting insulin and glucose metabolism. Excessive iNOS production is involved in the occurrence and progression of insulin resistance and CV diseases (CVD). This study aimed to assess the *in vivo* impact of IGF-1 on the activity and gene expression of iNOS in the hearts of obese rats, through the extracellular signal-regulated kinase 1/2 (ERK1/2) signaling pathway.

**Methods:**

Male adult Wistar rats were fed a standard (Control) or a high-fat (HF) diet for 12 weeks. After the 12^th^ week, half of the Control and HF rats received a single dose of IGF-1 (50 μg/kg, i.p.), while the other half was placebo-treated, and after 24 h the animals were euthanized.

**Results:**

The *in vivo* administration of IGF-1 led to a significant increase in nitric oxide (NO), iNOS gene and protein expression, endothelial nitric oxide synthase (eNOS) gene, ERK1/2, and nuclear factor kappa B (NFkB) levels in Control rats. In contrast, HF rats showed a decrease in NO, iNOS protein, and gene, eNOS gene, endothelin-1 and NFkB levels after IGF-1 treatment. Notably, the level of ERK1/2 in HF rats increased following IGF-1 treatment. These results underscore the significant impact of IGF-1 on iNOS activity in obese rat hearts.

**Discussion:**

Our findings suggest that the treatment of obese rats with IGF-1 could have significant implications for cardiac function, particularly in the context of obesity, by regulating cardiac iNOS.

## 1 Introduction

Cardiovascular (CV) diseases (CVD) are progressively rising, establishing them as the most widespread health problem globally. The significance of insulin-like growth factor 1 (IGF-1) in preserving CV health and the importance of IGF-1 for maintaining CV health have been brought to light by numerous studies (1–3). Cardiomyocytes locally produce IGF-1, a key player in CV health. It plays a crucial physiological role in the heart, promoting cardiomyocyte survival and functional adaptation (4). The effects of IGF-1 on the CV system (CVS) are extensive, including the maintenance of cellular homeostasis, regulation of vascular vasoconstriction/vasodilatation, cardiac apoptosis and autophagy, and inflammatory response (5, 6). IGF-1 has also been shown to enhance cardiac cells, improving cardiac performance (7). It exerts anti-inflammatory and anti-oxidant effects on the vasculature, reducing atherosclerotic plaque burden (8). Moreover, IGF-1 exhibits angiogenic properties by modulating endothelial junction protein levels and promoting angiogenesis in the vasculature (9). The association of IGF-1 deficiencies with CV aging underscores its protective effects against age-related CV alterations (10). This finding has significant clinical implications, suggesting that IGF-1 could be a potential therapeutic target for age-related CVD. IGF-1 promotes vasodilation by regulating the activity of endothelial nitric oxide synthase (eNOS). This enzyme synthesizes nitric oxide (NO), which relaxes blood vessels and improves blood flow (11). Additionally, IGF-1 enhances inducible NOS (iNOS) activity, contributing to vasodilation (12). IGF-1 positively influences cardiac function: it improves contractility, cardiac output, ejection fraction, and stroke volume. After a myocardial infarction, IGF-1 stimulates tissue remodeling and contractility, aiding in heart function recovery (1). Furthermore, IGF-1 impacts metabolic homeostasis by lowering insulin levels, increasing insulin sensitivity, and promoting glucose metabolism (13).

Obesity is linked to a reduction in the ability of blood vessels to dilate in response to the endothelium, which is a key indicator of impaired functioning of the inner lining of blood vessels, increasing the likelihood of developing various CVDs (14). In normal physiological conditions, the main source of NO production in the CVS is eNOS (15, 16). However, in pathophysiological conditions like obesity, there is an excessive production of NO due to the activation of iNOS, which leads to reduced heart contractility and has harmful effects on the heart (17). The evidence suggests that excessive production of iNOS has a role in the development of insulin resistance and CVD. It is believed to be the crucial connection between metabolic diseases associated with obesity and inflammation (18, 19). In the pathophysiology of heart failure with preserved ejection fraction (HFpEF), iNOS has been identified as a potentially crucial player, particularly in obesity and metabolic syndrome. In the myocardium of HFpEF patients and experimental animal models, elevated iNOS activity led to increased nitrosative stress and systemic inflammation via suppression of the IRE1α–XBP1 pathway (20). Another study showed that both short-term and long-term inhibition of iNOS in heart tissue of mice attenuated the HFpEF phenotype by reducing Akt S-nitrosylation and improving of mitochondrial function (21). Additionally, in the cross-sectional pilot study, HFpEF patients showed significantly higher serum levels of iNOS, indicating elevated nitrosative stress (22). These findings suggest that iNOS and related pathways may offer therapeutic potential. Elevated cardiac iNOS expression was also detected in rats and rabbits fed a high-fat (HF) diet, as well as spontaneously hypertensive rats (23–26).

The regulation of iNOS expression is mainly at the transcriptional level, with nuclear factor kappa B (NFκB) being the key transcription factor (27, 28). IGF-1 signaling activates NFκB (29). In addition, IGF-1 also interacts with intracellular kinases and extracellular signal-regulated kinases 1/2 (ERK1/2) (30). When ERK1/2 is activated, it subsequently phosphorylates and activates signaling molecules like NFκB (31). Obesity and insulin resistance modify NFκB and ERK1/2 activity (32) through increased oxidative stress and proinflammatory signalling (32). Elevated free fatty acid (FFA) levels stimulate the production of reactive oxygen species (ROS), activating NFκB, which further promotes the production of proinflammatory cytokines like TNF-α and IL-6. Additionally, FFAs and inflammation activate ERK1/2, which contributes to endothelial dysfunction associated with insulin resistance and obesity (32). However, earlier studies have shown decreased ERK1/2 phosphorylation in hypertrofied heart, which may be explained due to cyclic activation and deactivation of ERK1/2 (33, 34).

Obesity and CVD are linked to enhanced endothelin (ET)-1 production and activity (14). ET-1, a potent regulator of vascular tone, is produced in many tissues, including endothelial cells and cardiac myocytes (35), and together with NO, affects cardiomyocyte contractility through paracrine signaling (36). ET-1 is considered a potential risk marker for CVD and HfpEF, since its role has been pronounced in hypertension, coronary artery disease, atherosclerosis, myocardial infarction, etc. Additionally, ET-1, which originates from endothelial cells, plays a crucial role in endothelial dysfunction, a key pathophysiological component of CVD development and clinical progression (37–39). In addition, ET-1 promotes vasoconstriction, increases oxidative stress, reduces NO bioavailability and activates pro-inflammatory signaling pathways (40). Recent studies have implicated IGF-1 receptor transactivation in transducing the ET-1-induced signaling responses in the vasculature (9). ET-1-induced signaling is characterized by a rapid induction of the NFκB p65 subunit (41). ERK1/2 activity is involved in ET-1 expression, but on the other hand, ET-1 potently stimulates ERK1/2 (42).

In addition, IGF-1 is not only involved in cardiomyocytes survival but drives physiological, also known as adaptive, cardiomyocytes hypertrophy (43). Activation of the signaling pathway that includes IGF1R is also activated through physical activity and plays an important role in heart protection (44). In this case, the contractile function of the heart is preserved, with enhanced mitochondrial function and low apoptosis compared to pathological hypertrophy (7, 44, 45).

This study investigates the *in vivo* effects of IGF-1 on iNOS activity and gene expression in the hearts of obese rats, as well as the potential changes in two critical vasoactive factors, NO and ET-1. The research provides important insights into the relationship between IGF-1, obesity, and its effects on the heart, with potential implications for the development of new therapeutic strategies. Furthermore, we examined the effect of these changes on NFκB and ERK1/2 in the IGF-1-regulated expression of cardiac iNOS in obese rats. Our study tested the hypothesis that, *in vivo*, IGF-1 reduces the impact of obesity on cardiac iNOS activity by modulating ERK1/2/NF−κB signaling pathway.

## 2 Materials and methods

### 2.1 Animals and experimental treatment

The experiment was carried out at the Institute of Nuclear Sciences Vinca (Belgrade) on adult male Wistar rats (150-200 g). Animals (n=28) were divided into two groups. For 12 weeks, one group (n=14) was on a balanced diet for laboratory rats (labeled as CONT), while the other group (n=14) was on a high-fat (HF) diet (enriched with 42% fat) (labeled as HF) Nutritional composition and energy values of standard diet mixture for laboratory rats is presented previously (68). This experimental rat model was chosen based on literature data and our previous results (19, 46). The conditions where rats were bred included a 12:12 h light/dark cycle at 22 ± 2°C, with food and water available *ad libitum*. After the 12th week, half of the rats from both groups received intraperitoneal treatment with a single dose of human recombinant IGF-1 (Sigma-Aldrich, I3769-50UG) dissolved in saline (50 μg/kg) and were designated as IGF-1 and HF+IGF-1 groups. The specific dosage of IGF-1 was selected based on prior investigations (47–49). Since we opted to examine the molecular and signaling activity of IGF-1, the use of 50 µg/kg of IGF-1 represents a dose between efficacy and safety, making it a rational choice for experimental use. Simultaneously, the remaining half of the CONT and HF group rats were injected with an equivalent saline solution labeled CONT and HF. Animals were euthanized 24 hours after treatment, following 12 hours of overnight fasting, under deep anesthesia induced by a combination of ketamine and xylazine (80 mg/kg of ketamine [VetViva Richter GmbH, Austria] and 12 mg/kg of xylazine [VET-AGRO Multi Trade Company Sp. z o.o., Poland]). For the collection of blood samples, commercial vacutainers intended for biochemical analyses were used, which contain a factory-added anticoagulant, EDTA. Blood samples were collected from each animal through cardiac puncture, and plasma was isolated and stored at -20°C for subsequent analysis to determine the concentration of nitrite/nitrate (NO_2_^-^/NO_3_^-^). After excision, the hearts of all animals were stored at -80°C until further experiments were performed. The official Vinca Institute’s Ethical Committee for Experimental Animals (Veterinary Directorate – No. 323-07-02710/2017-05) approved all animal procedures and experimental protocols.

### 2.2 Heart tissue lysate preparation

Homogenization of rat hearts was performed on ice using an Ultra-turrax homogenizer (IKA-Werke GmbH & Co. KG) in a buffer solution (50 mM Tris, 150 mM NaCl, 2 mM EDTA, 10% glycerol, 1% Triton X-100, pH 7.4), supplemented with a cocktail containing protease (Complete ULTRA protease inhibitor cocktail tablets, Roche, Mannheim, Germany) and phosphatase inhibitors (PhosStop, Roche, Mannheim, Germany), along with an additional 2 mM sodium orthovanadate. The homogenates were then incubated for 1 hour at 4°C with constant rotation, followed by centrifugation at 100,000 x g for 20 minutes at 4°C to obtain supernatants. The protein concentrations of the supernatants were assessed using the bicinchoninic acid assay, employing a series of bovine serum albumin (BSA) solutions of known concentrations (from 0.1 μg/μl to 0.8 μg/μl) as the standard. The results were expressed as µg/µl. Until further analysis, all samples were stored at -80°C.

### 2.3 Measurement of NO_2_^-^/NO_3_^-^ concentrations

The plasma and heart lysate NO_2_^-^/NO_3_^-^ concentrations were determined using a nitrate/nitrite colorimetric assay kit (Cayman Chemical, Ann Arbor, MI, USA) following the manufacturer’s instructions. Heart protein lysates contained equal amounts of total proteins (500 μg) per sample. The concentrations of nitrite/nitrate were expressed in µmol/L.

### 2.4 SDS-PAGE and Western blot analysis

Total protein lysates extracts, containing equal amounts of total proteins (50 μg/lane), underwent either 8% or 10% SDS polyacrylamide gel electrophoresis (Bio-Rad Mini-Protean 3 Cell electrophoresis, Bio-Rad Laboratories, Hercules, CA, USA) (50), followed by transfer to polyvinylidene difluoride (PVDF) membranes, as per established protocols (51–53). The BSA (5%) was used for membrane blocking. Membranes were probed with antibodies targeting iNOS, eNOS, ET-1, total ERK1/2, p-ERK1/2 (Thr^202^/Tyr^204^), total NFkB-p65 and phosphorylated NFkB-p65 (Ser^536^) (**Supplementary Table S1**. Specification of antibodies). Subsequently, membranes were washed and incubated with an appropriate anti-rabbit HRP-conjugated secondary antibody. After further washing, the membranes were subjected to detection using an Enhanced Chemiluminescence kit (Immobilon Western Chemiluminescent HRP Substrate, Millipore). To ensure uniform protein loading of all samples, a mouse anti-β-actin monoclonal antibody was used, followed by an appropriate secondary anti-mouse HRP-conjugated antibody. Using Image J 1.45s software (National Institutes of Health, USA), densitometry analysis of the protein bands on X-ray film or membranes was performed.

### 2.5 Quantitative real-time PCR

Total RNA from cardiac tissue was extracted using Trizol reagent (Invitrogen Life Technologies, Paisley, GB), which followed manufacturer recommendations. The BioSpec-nano-Spectrophotometer (Shimadzu, USA) was used to determine the concentration and purity of RNA. cDNA synthesis assay was performed as previously described (25). Detailed information on primers used in qPCR (Applied Biosystem 7500, Carlsbad, USA) analysis is presented in **Supplementary Table S2**. The thermal cycle conditions for iNOS were 95°C for 4 min followed by 40 cycles that were run for 15s at 95°C and for 1 min at 61°C (25), while the thermal cycle conditions for eNOS were 95°C for 3 min followed by 40 cycles that were run for 15s at 95°C and for 32s at 58° C. The level of iNOS and eNOS gene expression was adjusted to the appropriate gene expression of *β-Actin*. Utilizing the 2^−ΔΔCt^ method, the relative quantification of mRNA expression was conducted (54).

### 2.6 Statistical analysis

Data were analyzed using Statistical Package for the Social Sciences 18.0 (SPSS Inc., Chicago, Illinois) and Excel Analysis. To ensure the reliability of our findings, we chose appropriate methods and samples and conducted the research very carefully and consistently. Additionally, to ensure the accuracy of our data, we take special care while preparing, importing, and handling the data in Excel and SPSS data sheets by continuously monitoring the data for accuracy and checking all datasets for duplicates and errors. Furthermore, we also performed data validation by using a data validation tool in Excel, where possible, verifying that the data conforms to the expected format and range to ensure it is suitable for further processing and analysis. The normality of the data was evaluated using the Shapiro-Wilk test, while the homogeneity of variances was assessed using Levene’s test. The Shapiro-Wilk test was not significant (p>0.05), indicating that all the data we obtained are normally distributed. Levene’s test was significant (p<0.05) only for the data for ERK protein phosphorylation level, indicating that the variances between the groups are significantly different, while for all other results Levene’s test was not significant (p>0.05), indicating that the variances between the groups are not significantly different. Descriptive statistical methods were used to present data as mean ± SEM. Depending on the Levene’s test results, differences between CONT and IGF-1, CONT and HF group, and HF and IGF-1+HF group were analysed by performing One-way ANOVA tests followed by Fisher’s Least Significant Difference (LSD) *post hoc* test for multiple comparisons (for concentrations of NO_2_-/NO_3_- in plasma and heart lysate, iNOS, eNOS, and ET-l protein level, p65 NFkB phosphorylation level, and iNOS and eNOS mRNA expression) or Welch’s ANOVA followed by Games-Howell test (for ERK protein phosphorylation level). Differences between CONT and IGF-1, CONT and HF group, and HF and IGF-1+HF group were presented with significance set at p<0.05.

## 3 Results

### 3.1 The influence of IGF-1 on the concentrations of NO_2_^-^/NO_3_^-^ in plasma and heart lysate

**Figure 1:**
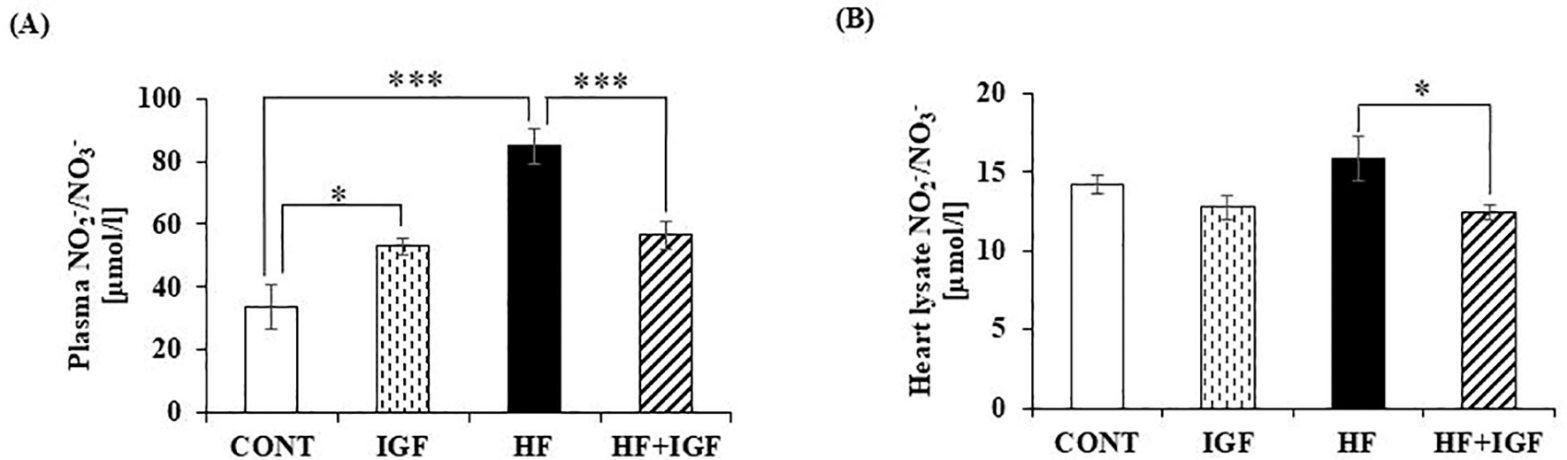
Effects of IGF-1 on plasma **(A)** and heart lysate **(B)** NO_2_^-^/NO_3_^-^ concentrations **(A)** Plasma NO_2_^-^/NO_3_^-^ concentrations are expressed in µmol/l and represent mean ± SEM, (n=5-6) *p<0.05 (IGF-1 *vs* CONT), ***p<0.001 (HF *vs* CONT). **(B)** Heart lysate NO_2_^-^/NO_3_^-^ concentrations are expressed in µmol/l and represent mean ± SEM, (n=5-6) *p<0.05 (HF+IGF-1 *vs* HF). CONT-control group, IGF-1 - IGF-1-treated group, HF – high-fat group, HF+IGF-1 - IGF-1-treated high-fat group.

Nitric oxide is important in cardiac function (55). However, in cases of obesity, either a deficiency or excessive production of NO can lead to detrimental consequences on the heart (56). To evaluate the *in vivo* effect of IGF-1 treatment on NO generation, we assessed the NO_2_^-^/NO_3_^-^ concentrations in rats’ plasma and heart lysate. Plasma concentration of NO_2_-/NO_3_- in HF-fed animals was significantly increased compared to control animals (p<0.001) (**Figure 1A**), while in heart lysate, obesity increased the level of NO_2_-/NO_3_-, but this increase was not significant (**Figure 1B**). When comparing the plasma concentration of NO_2_^-^/NO_3_^-^ with the corresponding controls, the concentration of NO_2_^-^/NO_3_^-^ in IGF-1-treated control rats was significantly elevated (p<0.05), whereas, in HF+IGF-1 rats, it was significantly lower (p<0.001) (**Figure 1A**). The NO_₂_⁻/NO_3_⁻ concentration in heart lysates was unchanged in IGF-1-treated control rats, but significantly decreased in our HF-fed rats treated with IGF-1 (p<0.05) (**Figure 1B**).

### 3.2 IGF-1 effects on iNOS and eNOS protein levels and iNOS and eNOS mRNA expression in the heart of rats

**Figure 2:**
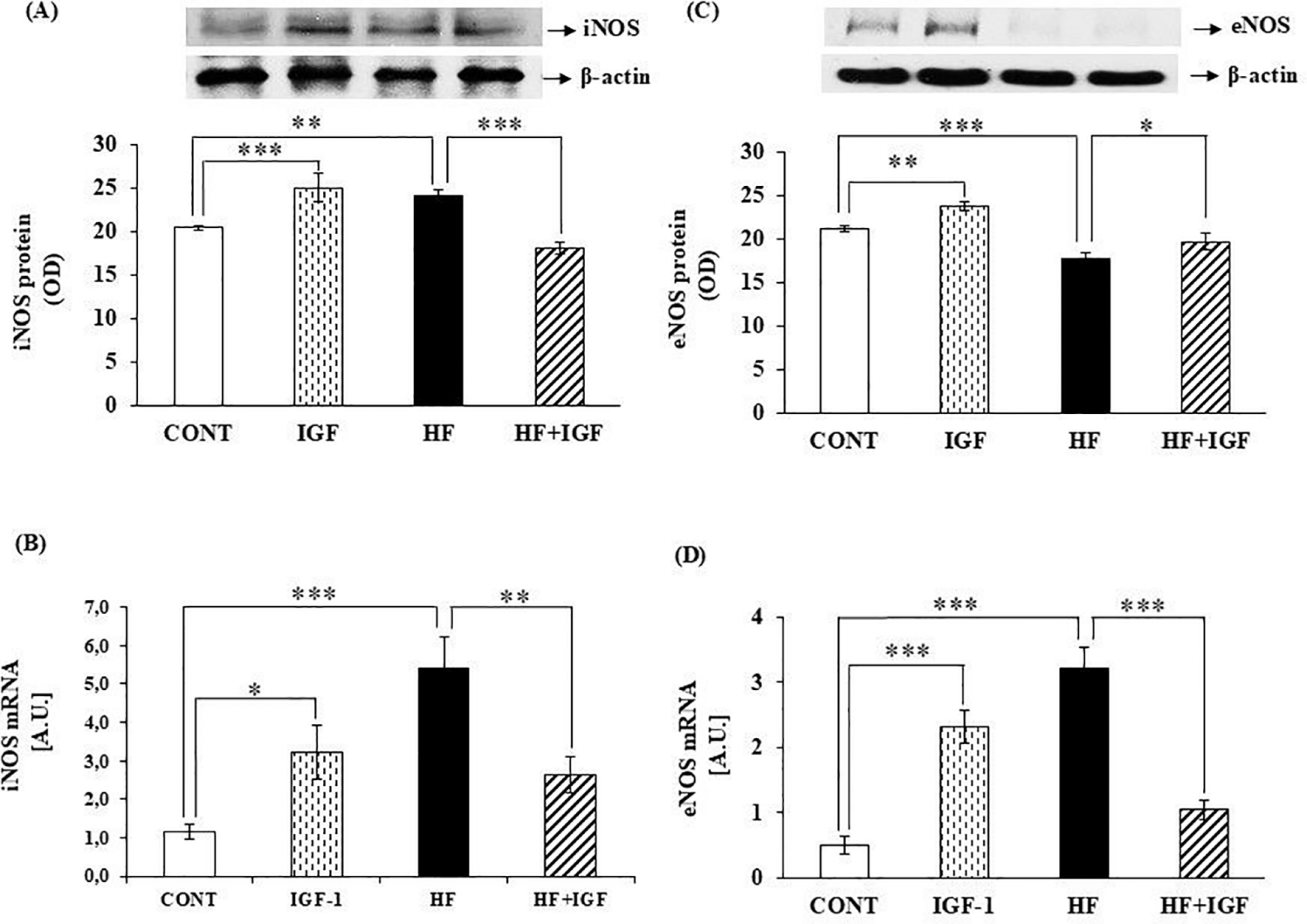
Effects of IGF-1 on iNOS and eNOS protein level and iNOS and eNOS mRNA expression in the heart of rats **(A)** Protein expression of iNOS is presented as OD (n=5-7). **(B)** mRNA expression of iNOS (expressed as A.U., n=4-5). **(C)** Protein expression of eNOS is presented as OD (n=5-7). **(D)** mRNA expression of eNOS (expressed as A.U., n=4-6). CONT-control group, IGF-1 - IGF-1-treated group, HF – high-fat group, HF+IGF-1 - IGF-1-treated high-fat group, A.U. – arbitrary units, OD – Optical Density. *p<0.05 (IGF-1 vs CONT, HF+IGF-1 vs HF), **p<0.01 (IGF-1, HF vs CONT. HF+IGF-1 vs HF), ***p<0.01 (IGF-1, HF vs CONT, HF+IGF-1 vs HF).

Given that obesity is defined by an accumulation of fat in many tissues, leading to long-term low-level inflammation that could trigger the production of iNOS, we conducted a further study to examine the impact of IGF-1 on the levels of iNOS protein and to delineate the molecular mechanisms that underlie the IGF-1 effects on gene transcription in the heart. Obesity itself significantly increased the level of iNOS protein (p<0.01) (**Figure 2A**) and iNOS mRNA expression (p<0.001) (**Figure 2B**) compared with control animals. The levels of cardiac iNOS protein were markedly higher (p<0.001) in control rats treated with IGF-1 compared with control rats. Moreover, our findings indicate that administering IGF-1 significantly reduces cardiac iNOS protein levels (p<0.001) in HF-fed rats compared with HF-fed rats who did not receive treatment (**Figure 2A**). Similarly, the cardiac iNOS mRNA expression level is elevated (p<0.05) in IGF-1-treated control rats compared with non-treated control rats. At the same time, IGF-1 treatment significantly decreases cardiac iNOS mRNA expression (p<0.01) in HF-fed rats compared with non-treated HF-fed rats (**Figure 2B**). These data suggest that IGF-1 may affect iNOS gene transcription. Since NO, which performs biological functions in the myocardium, is mainly produced by eNOS, we further examined the *in vivo* effects of IGF-1 on cardiac eNOS mRNA levels. The results show that obesity significantly decreased (p<0.001) the level of eNOS protein (**Figure 2C**), while significantly increasing (p<0.001) the level of eNOS mRNA expression (**Figure 2D**). *In vivo* treatment with IGF-1 elevates the level of cardiac eNOS protein (p<0.01) (**Figure 2C**) and eNOS mRNA expression levels (p<0.001) (**Figure 2D**) in control rats compared with non-treated control rats. On the other hand, treatment of HF-fed animals with IGF-1 increased (p<0.05) the level of eNOS protein (**Figure 2C**) and decreased (p<0.001) the level of cardiac eNOS mRNA (**Figure 2D**) compared with obese animals.

### 3.3 Effects of IGF-1 on the expression of ET-1 in the heart of rats

**Figure 3:**
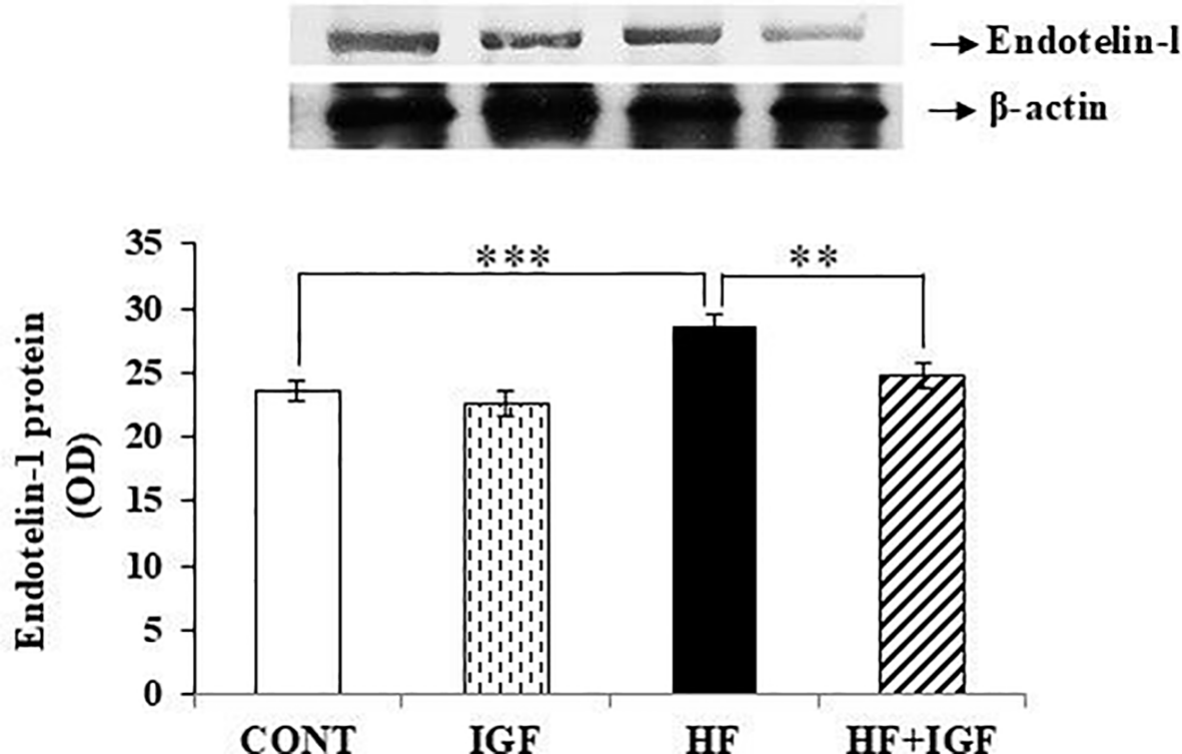
Effects of IGF-1 on the expression of Endotelin-1 in the heart of rats Protein expression of total Endotelin-1 is presented as OD (n=6-7). IGF-1 - IGF-1-treated group, HF – high-fat group, HF+IGF-1 - IGF-1-treated high-fat group, OD – Optical Density. ***p<0.001 (HF vs CONT), **p<0.01 (HF+IGF-1 vs HF).

To gain more insight into IGF-1 vasodilatory effects, we next examined ET-1 protein expression in the heart lysate fraction. Endothelin-1 is a strong vasoconstrictor, and together with NO regulates vascular tone and affects cardiomyocyte contractility (57). Obesity increases expression of ET-1 mRNA in the heart, which contributes to cardiovascular complications (58). We also found that cardiac ET-1 protein level was significantly increased (p<0.001) in HF-fed animals compared to control animals. In addition, our results indicate that IGF-1 treatment did not change the level of ET-1 protein expression in control rats, but decreased the expression of ET-1 (p<0.01), and thereby normalized its level in the HF group, compared with HF non-treated rats (**Figure 3**).

### 3.4 Effects of IGF-1 on cardiac ERK1/2 phosphorylation

**Figure 4:**
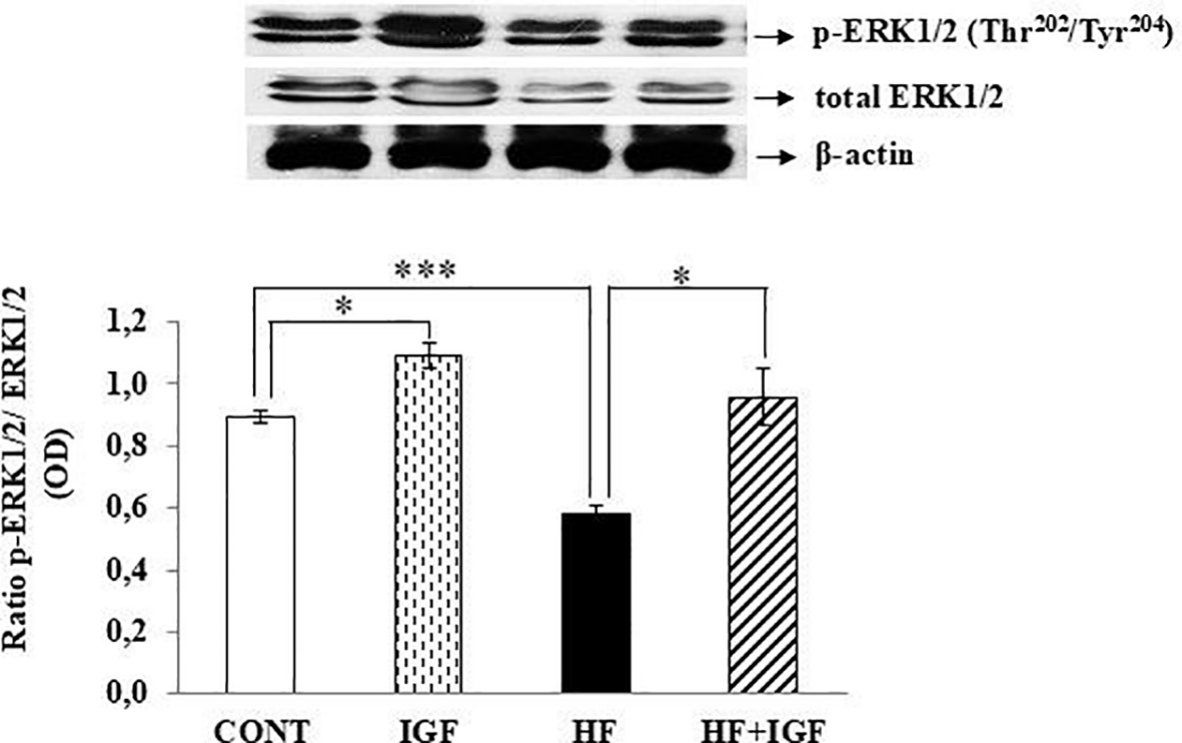
Effects of IGF-1 on cardiac ERK1/2 phosphorylation. The results were obtained by the ratio of ERK1/2 phosphorylated on Thr^202^/Tyr^204^ (p-ERK1/2) and total ERK1/2. Phosphorylation and expression results are presented as OD (n=5-6). CONT-control group, ERK1/2 - extracellular signal-regulated kinase 1/2, IGF-1 - IGF-1-treated group, HF – high-fat group, HF+IGF-1 - IGF-1-treated high-fat group, OD – Optical Density. *p<0.05 (IGF-1 *vs* CONT), ***p<0.001 (HF *vs* CONT), *p<0.05 (HF+IGF-1 vs HF).

Since IGF-1 has been found to promote ERK1/2 activity *in vitro* in vascular smooth muscle cells (59, 60), we investigated whether the regulatory mechanisms of IGF-1 activation of ERK1/2 *in vivo* are similar. In this study, we examined the modulation of cardiac iNOS under IGF-1 treatment in control and obese rats by quantifying the phosphorylation levels of ERK1/2. The findings show that obesity significantly decreases the density ratio of phospho (Thr^202^/Tyr^204^) to total forms of ERK1/2 (p<0.001), while the administration of IGF-1 resulted in an elevated density ratio of phospho (Thr^202^/Tyr^204^) to total forms of ERK1/2. This effect of IGF-1 was observed in both the control group (p<0.05) and the group of HF-fed rats (p<0.05), as compared with their respective untreated control groups (**Figure 4**).

### 3.5 Effects of IGF-1 on cardiac p65 NFkB phosphorylation

**Figure 5:**
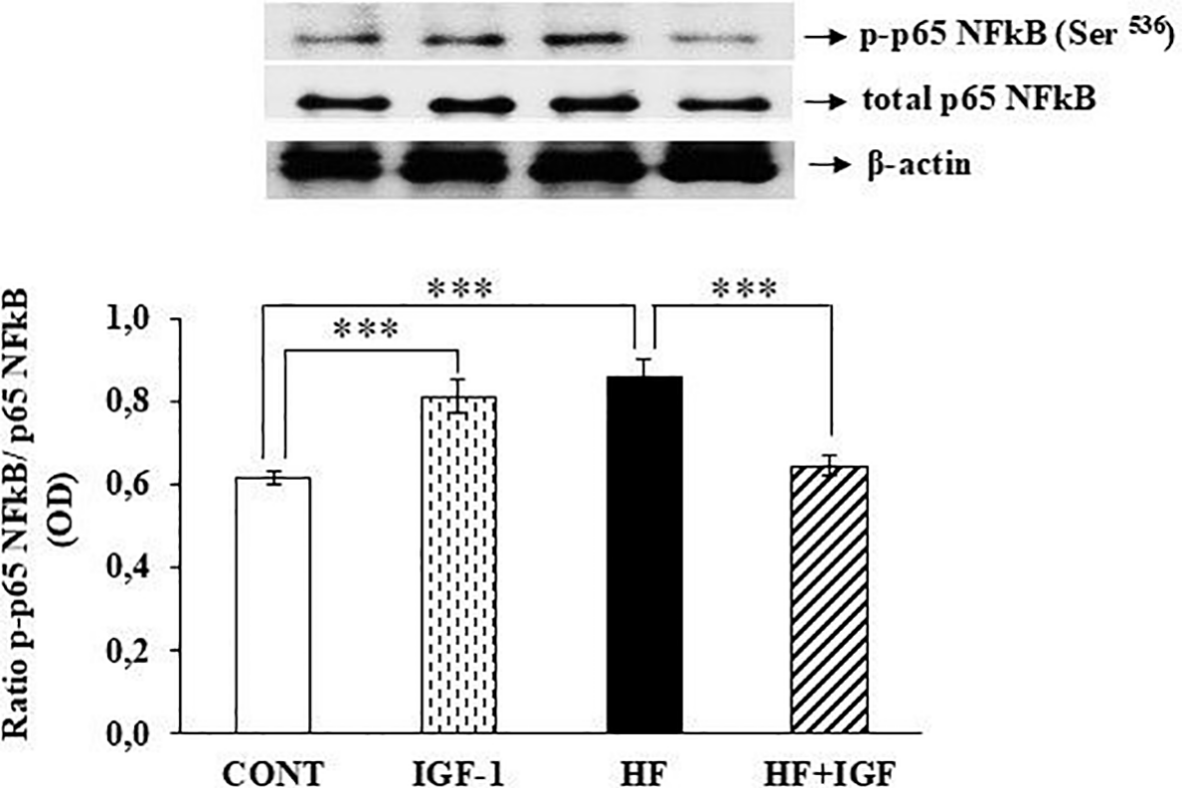
Effects of IGF-1 on cardiac p65 NFkB phosphorylation. The results were obtained by ratio of p65 NFkB phosphorylated on Ser^536^ (p-p65 NFkB) and total p65 NFkB. Phosphorylation and expression results are presented as OD (n=7). CONT-control group, IGF-1 - IGF-1-treated group, HF – high-fat group, HF+IGF-1 - IGF-1-treated high-fat group, OD – Optical Density, NFkB - nuclear factor kappa-light-chain-enhancer of activated B cells. ***p<0.001 (IGF-1, HF *vs* CONT, HF+IGF-1 vs HF).

Because the control of iNOS expression mainly occurs at the transcriptional level, with NFκB playing a crucial role as a transcription factor (27, 28), we conducted additional research to investigate the mechanism of cardiac iNOS regulation in control and obese animals treated with IGF-1. This was done by assessing the phosphorylation level of the p65 NFkB subunit. Obesity increased the phosphorylation level of the p65 NFkB subunit on Ser^536^ (p<0.001) compared with control animals. The phosphorylation level of the p65 NFkB subunit on Ser^536^ is significantly higher in the heart of IGF-1-treated control rats (p<0.001) than in untreated control rats. In contrast, the administration of IGF-1 resulted in a notable reduction (p<0.001) in the phosphorylation level of the p65 NFkB subunit, on Ser^536^ in HF rats compared with HF rats that did not receive IGF-1 (**Figure 5**).

## 4 Discussion

Obesity, a condition linked to chronic, low-grade inflammation (61), has detrimental effects on CV function, leading to impaired vasodilation and endothelial dysfunction (62). The extensive literature underscores the positive effects of NO on the CVS (63, 64), with a particular focus on obesity’s impact on iNOS expression and NO production (65, 66). Alterations in serum IGF-1 levels are associated with the progression of CVD (10). By regulating iNOS and two critical vasoactive factors, NO and ET-1, potentially through the modulation of ERK1/2 and NF-κB signaling pathway, IGF-1 demonstrates significant benefits. This study shows the promising potential of IGF-1 as a therapeutic strategy for CVD in obesity. We have previously shown in the same rats that an HF diet significantly increases body mass and serum glucose concentration in both HF groups of rats compared with their controls, while IGF-1 treatment had no effect on body mass or glucose concentration (67, 68). Also, we previously showed on the same rats that IGF-1 administration has cardioprotective effects in obesity due to attenuation of cardiac hypertrophy (68). The findings of this study represent a novel approach to understanding the effects of IGF-1 on the heart of obese rats. We assessed the protein and gene expression of iNOS in the cardiac tissue of rats to investigate the impact of IGF-1 on iNOS regulation. The analysis indicated a significant increase in control rats’ cardiac iNOS protein and mRNA levels. In contrast, these levels were reduced in the hearts of obese rats treated with IGF-1 compared with those of untreated rats. This data highlighted IGF-1 as a significant regulator of iNOS regulation. Consistent with our findings, previous *in vitro* studies conducted by our group and others have demonstrated that IGF-1, under physiological conditions, stimulates iNOS expression in vascular smooth muscle cells (59) and cardiomyocytes (69). IGF-1 treatment and the resulting IGF-1-dependent signaling enhance tetrahydrobiopterin (BH_4_) production in the heart (70), suggesting that IGF-1 facilitates the recruitment of iNOS for NO generation. On the other hand, Serhan et al. (71) found that systemic IGF-1 treatment in male Wistar rats reduces iNOS mRNA expression in the ischemic hemisphere of the brain following induced stroke. This study represents the first *in vivo* investigation into the response of the heart’s iNOS to IGF-1 and its associated signaling within a physiological and pathophysiological framework. IGF-1 demonstrates beneficial effects in the hearts of obese rats by normalizing the expression levels of the iNOS gene and protein. Prolonged activation of iNOS results in elevated levels of NO, which can be detrimental as excessive NO concentrations may lead to oxidative stress, inflammation, and tissue damage (72, 73). Our previous research indicated elevated NO_2_^-^/NO_3_^-^ levels in the hearts of obese rats (65). Subsequent experiments measured NO concentration in the heart and plasma of control and obese rats administered IGF-1. The elevated plasma levels of NO_2_^-^/NO_3_^-^ in control IGF-1-treated rats, alongside the reduced plasma and heart lysate concentrations of NO_2_^-^/NO_3_^-^ in HF-fed rats receiving IGF-1, correspond with alterations in iNOS expression. These findings suggest that IGF-1 treatment in obesity attenuates disturbed NO signaling and may have an anti-inflammatory and cardioprotective role. This finding provides unique insights into the potential therapeutic effects of IGF-1 on CVD associated with obesity.

Furthermore, the stimulation of NO production by IGF-1 may also result from eNOS activity (74–76). IGF-1 inhibited atherosclerosis, a chronic inflammatory condition, in ApoE-/- mice subjected to a HF diet for 12 weeks (77). This effect correlated with elevated circulating NO levels and increased vascular eNOS expression (77). IGF-1 treatment of our control animals increased eNOS expression, which is in correlation with our group’s previous investigation of rat aortic endothelial cells, which showed that treatment of rat aortic endothelial cells with IGF-1 increased eNOS activity (78). Treatment of hypophysectomized female rats with IGF leads to increased expression of eNOS in the intima layer of the aorta (79). In obese rats, IGF-1 decreased mRNA eNOS level, while the level of eNOS protein was elevated, indicating possible posttranscriptional or translational mechanisms of regulation. These findings suggest potential directions for future research, such as investigating the specific mechanisms by which IGF-1 regulates eNOS activity and the possible therapeutic implications of these mechanisms for CVD associated with obesity.

Our investigation into the expression of the vasoconstrictor molecule ET-1 further explores the effects of IGF-1 on the detrimental impact of obesity on the heart. ET-1 has been shown to reduce vascular NO bioavailability, contributing to the pathophysiology of hypertension (35). Obesity induces inflammation and oxidative stress (80), leading to increased production of peroxynitrite ions, which contribute to the vasoconstrictive effects of ET-1 (8, 81). ET-1 regulates vascular tone and cardiac function (75). The increase in ET-1 levels due to obesity is linked to multiple CVDs, including hypertension, chronic heart failure, and coronary artery disease (14). Our findings demonstrate the encouraging potential of IGF-1 to reduce ET-1 protein expression in the hearts of both control and obese rats, suggesting vasoprotective effects of IGF-1 in cardiac tissue. Increased expression of the ET-1 gene in the aorta and elevated systolic blood pressure were noted in IGF-1-deficient mice (82). Furthermore, reduced IGF-1/IGF-1R expression and diminished protective actions have been observed in subclinical hypertensive arterial injury (83). Results from this study also align with our previously obtained results where IGF treatment decreases serum levels of angiotensin II (Ang II) and AT1R, while increasing the level of AT2R protein expression in the heart of obese animals (68). Our results indicate that IGF-, by decreasing the level of ET-1, in addition to the already observed decrease in Ang II, AT1R, and increase of AT2R, may have protective effects on the heart, potentially through vasodilatory and anti-inflammatory mechanisms. These findings suggest that IGF-1 could be a potential therapeutic target for treating CVD associated with obesity, and further research in this area could lead to the development of novel treatment strategies.

We also looked at the role of ERK1/2 and NFκB signaling pathways to better understand the molecular mechanism of IGF-1 control of iNOS production. ERK1/2 plays a significant role in the pathophysiology of the heart in obesity (84). Activated ERK1/2 phosphorylates several downstream signaling molecules, including transcription factors (85), leading to the regulation of iNOS (86). iNOS is recognized as a target gene of NF-κB (87). NFκB plays a crucial role in mediating iNOS gene expression in response to inflammation, a common characteristic of obesity (87). IGF-1 activates the ERK1/2 pathway (88) and modulates NFκB activity (89, 90). The interaction between ERK1/2 signaling and NFκB phosphorylation has been demonstrated (85). Our findings imply that IGF-1 treatment increased ERK1/2 phosphorylation in control rats, which aligns with an observed increase in p65 NFκB subunit phosphorylation and an apparent increase in iNOS gene and protein expression. On the other hand, in obese rats, IGF-1 treatment enhanced the phosphorylation of cardiac ERK1/2 while reducing the phosphorylation of p65NFκB and the expression levels of iNOS mRNA and protein. Interestingly, IGF−1 treatment increases ERK1/2 phosphorylation in both control and obese rats, but has opposite effects on NF-κB/iNOS depending on metabolic status. Previous *in vitro* studies showed that IGF-1 is a potent stimulator of ERK 1/2 and NFκ-B/iNOS (91, 92), which was also observed in our study in physiological condition. However, opposite effects of IGF-1 on iNOS stimulation in hearts of obese rats were observed. This dual role of IGF-1 could be explained by the anti-inflammatory effect of IGF-1 in pathological conditions, which is achieved through reduced activation of NF-κB and consequently diminished iNOS level (93, 94). These findings support other investigations that IGF-1 influences the NFκB transcription factor by modifying the phosphatidylinositol 3−kinase/protein kinase B (PI3K/Akt) and mitogen-activated protein kinase (MAPK) signaling pathways (90). This suggests that another signaling pathway may be activated to regulate iNOS expression and activity in obese rats. Our published results show that IGF-1 treatment induced activation of IRS-1, as well as the downstream activation of the Akt signaling pathway (68). In the same study, increased phosphorylation of mTOR on Ser^2481^ was noted. The PI3K/Akt and ERK pathways can be regulated through different phosphorylation, which can influence NFκB activation (95). Thus, the crosstalk between ERK and NF-κB pathways in obesity may be changed through activation of PI3K/Akt/mTOR signaling. Modulation of these signaling pathways indicates complex signaling underlying the regulation of iNOS in the heart and requires further investigation. Furthermore, IGF-1 inhibits IkB kinase, suppresses IkB phosphorylation and degradation, and subsequently reduces the expression of inflammation-related genes (96). The suppression of NF-κB inhibited ET-1 expression and protected a rat model of acute respiratory distress syndrome (97). IGF-1 mitigates obesity-related vascular complications by suppressing NO and ET-1 levels, probably through signaling pathways that involve reductions of NF-κB phosphorylation and elevation of ERK1/2 phosphorylation.

Although IGF-1 is generally considered cardioprotective in pathological states, it is important to emphasize that chronically elevated IGF-1 in healthy myocardium may exert maladaptive effects, including pro-hypertrophic signaling and structural remodeling. These include pro-hypertrophic activation of the PI3K-Akt-p70S6K1 pathway and disruption of the tightly regulated balance between cardiomyocyte growth and metabolic demand (43). A study on patients with no known CVD showed that both low and high levels of circulating IGF-1 are associated with increased risk of CVD in the general population (98).

Despite important innovative findings, our study has some limitations. During the conducting of experiments within this study, the whole heart was used. In our future studies, we plan to analyze the effects of IGF-1 on the different cardiac segments, to determine whether tissue responses are region-specific. Also, while this study showed the potential of IGF-1 in regulating iNOS expression in obesity, further mechanistic studies are necessary to elucidate the molecular mechanisms of iNOS regulation under the influence of IGF-1 in more detail. Using inhibitors of MEK/ERK (U0126 - a MEK1/2 inhibitor that blocks ERK1/2 activation), PI3K/Akt (LY294002 and MK-2206), and NF-κB (BAY 11-7082 - an inhibitor of IκBα phosphorylation and NF-κB activation) will allow us to directly interrogate the functional role of ERK1/2, Akt, and NF-κB in mediating the IGF-1–induced effects on iNOS. This approach could demonstrate whether pharmacological blockade of one of these specific pathways can redirect the IGF-1 response from the maladaptive to the protective phase. Also, an innovative feature of the future planned experiments is that resistance to IGF-1 signaling in CV tissue may be equally important as resistance to insulin signaling due to the local autocrine/paracrine effects of IGF-1.

## 5 Conclusions

Our preliminary study suggests that administering IGF-1 to obese rats provides beneficial effects on their heart by controlling the activity of cardiac iNOS. This finding is particularly intriguing given the intricate relationship between IGF-1 and the signaling pathways that regulate inflammation and cellular response. We propose that IGF-1 reduces the vascular problems caused by obesity by suppressing the production of NO and ET-1 with the participation of the transcription factor NFκB. However, since the exact mechanism of this regulation is not yet fully described, further research is needed to shed light on the precise mechanism of action of IGF-1 on iNOS in the heart. This ongoing scientific exploration is crucial for advancing our understanding of cardiovascular health and obesity. The potential impact of this research on future treatments for obesity-related CV issues is significant. This study suggests an innovative approach and possible novel therapeutic strategies that could include applications of IGF-1 to diminish obesity-related CVD, thereby reinforcing the importance of our research in CV health and obesity.

## Data Availability

All relevant data is contained within the article. The original contributions presented in the study are included in the article/**Supplementary Material**, further inquiries can be directed to the corresponding author/s.
