# Supplementary material for: Insulin-like growth factor 1 (IGF-1)-induced changes in cardiac inducible nitric oxide synthase (iNOS) in obese rats
Source: Front Endocrinol (Lausanne). 2026 Jan 16;16:1716392. doi: 10.3389/fendo.2025.1716392 (PMC12855136; doi:10.3389/fendo.2025.1716392)
Supplement: Supplementary file 1 [file DataSheet1.pdf]

Supplementary material

Re: Insulin-like growth factor 1 (IGF-1)-induced changes in cardiac inducible nitric oxide synthase (iNOS) in obese rats

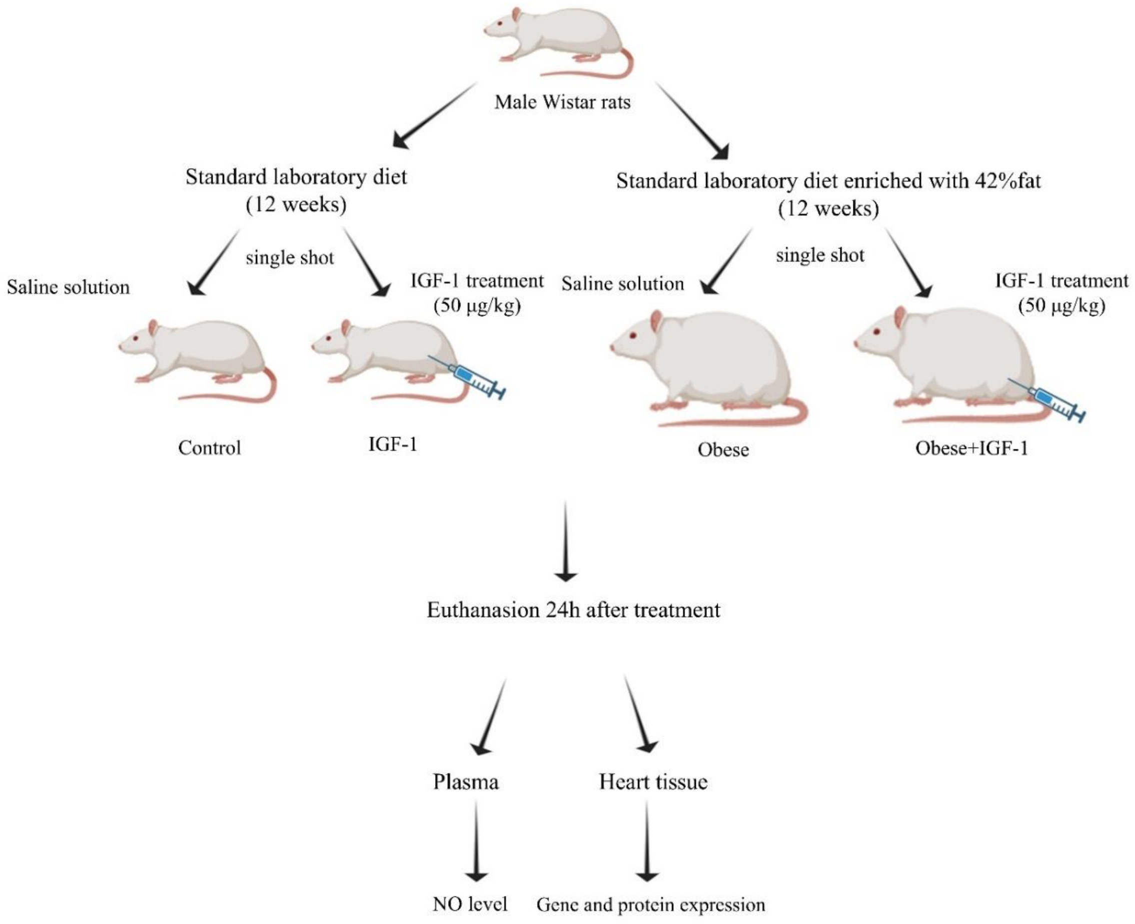

Figure S1. Experimental design

Table S1. Specification of antibodies

Antibody	Dilution	Cat. number	Manufacturer
Phospho-p44/42 MAPK (ERK1/2) (Thr^{202}/Tyr^{204})	1:1000	#9101	Cell Signaling
p44/42 MAPK (ERK1/2)	1:1000	#9102	Cell Signaling
iNOS	1:1000	ab15323	Abcam
Phospho-NF-kappaB p65 (Ser^{536})	1:1000	#3033	Cell Signaling
NF-kappaB p65	1:1000	#6956	Cell Signaling
Endothelin 1	1:1000	ab2786	Abcam
eNOS	1:1000	#32027	Cell Signaling
beta Actin	1:1000	sc-81178	Santa Cruz
AM-HRP	1:2000	sc-358914	Santa Cruz
AR-HRP	1:2000	HAF008	R&D Systems

Table S2. Sequences of primer pairs used in qPCR analysis

Rat gene	Primer		GenBank accession number	PCR product length (bp)
iNOS	forward	5′-AGAAGTCCAGCCGCACCAC-3′	NM_012611	103
	reverse	5′-TGGTTGCCTGGGAAAATCC-3′		
eNOS	forward	5′-TGGAAATTAACGTGGCTGTG-3′	NM_021838	112
	reverse	5′-GCCTTCTGCTCATTTTCCAA-3′		
β actin	forward	5′-CCCTGGCTCCTAGCACCAT-3′	NM_031144	76
	reverse	5′-GAGCCACCAATCCACACAGA-3′		

eNOS - endothelial nitric oxide synthase, iNOS - inducible nitric oxide synthase.
